# Tryptophan Dietary Impacts Gut Barrier and Metabolic Diseases

**DOI:** 10.3389/fimmu.2019.02113

**Published:** 2019-09-10

**Authors:** Soraya Taleb

**Affiliations:** Institut National de la Santé et de la Recherche Médicale (INSERM), Unit 970, Paris Cardiovascular Research Center, and Université Paris-Descartes, Paris, France

**Keywords:** tryptophan, indoleamine 2, 3-dioxygenase, gut microbiota, metabolic syndrome, cardiovascular disease and cardiometabolic diseases

## Abstract

The intestine has a major role in the digestion and absorption of nutrients, and gut barrier is the first defense line against harmful pathogens. Alteration of the intestinal barrier is associated with enhanced intestinal permeability and development of numerous pathological diseases including gastrointestinal and cardiometabolic diseases. Among the metabolites that play an important role within intestinal health, L Tryptophan (Trp) is one of the nine essential amino acids supplied by diet, whose metabolism appears as a key modulator of gut microbiota, with major impacts on physiological, and pathological pathways. Recently, emerging evidence showed that the Trp catabolism through one major enzyme indoleamine 2,3-dioxygenase 1 (IDO1) expressed by the host affects Trp metabolism by gut microbiota to generate indole metabolites, thereby altering gut function and health in mice and humans. In this mini review, I summarize the most recent advances concerning the role of Trp metabolism in host–microbiota cross-talk in health, and metabolic diseases. This novel aspect of IDO1 function in intestine will better explain its complex roles in a broad range of disease states where the gut function affects local as well as systemic health, and will open new therapeutic strategies.

## Introduction

The prevalence of Western diet-induced metabolic syndrome (MetS) is booming, affecting more than 2 billion people worldwide and accounting for at least 3 million deaths per year (1). This becomes worrying since MetS is the major contributor of the persistent increase in cardiovascular diseases (CVD), including myocardial infarction (MI), which is the main complication of atherosclerosis. Many patients with obesity suffer from adverse metabolic complications and associated atherosclerosis, whereas others remain “metabolically healthy obese” (MHO), although they still have a higher CVD risk than normal weight and metabolically healthy subjects (2). The inconsistency regarding individual susceptibility to cardiometabolic diseases is still an issue that is currently not sufficiently addressed. This susceptibility to cardiometabolic diseases is mainly associated with environmental factors such as diet. One link between environment and disease is gut microbiota and the disruption in host–microbiota cross-talk could be involved in disease pathogenesis. The intestinal epithelium is a single-cell layer that constitutes a physical barrier against the external entities due to the expression of epithelial tight and adherence junctions. It acts as a selectively permeable barrier permitting the absorption of nutrients such as amino acids, carbohydrates, lipids, electrolytes, and water, while avoiding pathogen invasion. The dysfunction of this barrier as observed in inflammatory diseases leads to enhanced permeability and translocation of microbial entities such as lipopolysaccharide (LPS) to systemic circulation, which may cause observed inflammation responsible for obesity complications (3). A recent study pinpoints toward the importance of hyperglycemia as an initial trigger responsible for the disruption of tight and adherence junctions leading to the observed increase in intestinal permeability related to MetS (4). However, other actors should be involved as an increase in intestinal permeability is also observed in other diseases without glycemia disruption such as intestinal bowel diseases (5). In this context, it is not clear whether the permeability changes are a primary event in the disease development or a secondary result elicited by intestinal inflammation.

The association between altered gut microbiota or dysbiosis, inflammation, and cardiometabolic diseases is becoming increasingly clear but remains poorly understood (6, 7). In the CVD context, the interplay between dietary composition and gut microbiota-derived metabolites has been highlighted by the discovery of the role of Trimethylamine *N*-oxide (TMAO) in promoting atherosclerosis (8). Besides, L tryptophan (Trp) intake has recently emerged as a potential link between altered gut microbiota, impairment of intestinal immunity and disease development (9).

In this mini review, I summarize current evidence supporting the involvement of Trp catabolism by both the host, and gut microbiota in the context of MetS. Furthermore, I describe the potential mechanisms of action of Trp metabolites in modulating the local intestinal homeostasis, which may impact systemic metabolic parameters.

## Tryptophan Catabolism in Cardiometabolic Diseases

**Figure 1:**
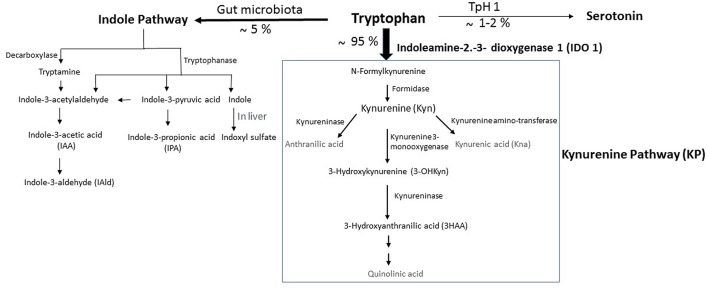
Simplified illustration of the kynurenine pathway in the gastrointestinal tract. Tryptophan (Trp) is predominantly converted into kynurenine (Kyn) pathway (KP) by the indoleamine 2,3-dioxygenase 1 (IDO). A small amount of Trp is converted by gut microbiota through the action of the enzyme tryptophanase, into indole, and its derivatives and into tryptamine. Indole metabolites could be converted in the liver into indoxyl sulfate. Another fraction of Trp is converted through Trp hydroxylase 1 (TpH 1) into serotonin.

Trp is one of nine essential amino acids brought by the diet, which the metabolism appears now as a key modulator of gut microbiota impacting major physiological, and pathological pathways (10, 11). In mammalian cells, Trp is primarily degraded through the kynurenine pathway (KP), a cascade of enzymatic steps leading to the generation of several biologically active compounds. Subsequent to Trp absorption via enterocyte transporters in the large intestine, Trp transits into the hepatic portal system where it is utilized by the liver for the KP through tryptophan 2,3-dioxygenase (TDO). Unused Trp is then secreted into the bloodstream and is available for use by peripheral tissues. The Trp degradation step in peripheral tissues is mainly due to indoleamine 2,3-dioxygenase (IDO)1, which contributes to the major Trp catabolism in extrahepatic tissues as compared with that resulting from IDO2 isoform. Specifically, Trp is degraded into *N*-formylkynurenine, leading to the generation of several active metabolites, including kynurenine (Kyn), 3-hydroxykynurenine (3-OHKyn), kynurenic acid (Kna), 3-hydroxyanthranilic acid (3HAA), and quinolinic acid. A small fraction of Trp is converted to serotonin and melatonin via the serotonin pathway, mainly in the gastrointestinal tract (Figure 1). During inflammation, IDO1 is up-regulated mostly in macrophages, and dendritic cells by proinflammatory stimuli, notably interferon (IFN)-γ (12). IDO1 exerts its biological effects mainly through the generation of downstream metabolites that suppress effector T-cell function, and favor the differentiation of regulatory T cells (Tregs) (13). However, IDO1 does not appear instrumental in these functions, as IDO1 knockout mice do not develop an autoimmune phenotype. This may be due to a compensatory or counter-regulatory mechanism (14). In addition, the biological effects of IDO1 may go beyond its role in the regulation of the immune response. Indeed, IDO1 activity was shown to contribute to arterial vessel relaxation and to the control of blood pressure in the context of septic shock (15). IDO1 activity was also shown to play a critical role in aneurysm development through favoring vascular smooth muscle cell (VSMC) apoptosis (16), and the increase of metalloproteinase (MMP)-2 expression in VSMC (17). On the other hand, metabolites generated from kynurenine may regulate diverse cellular functions, including viability (18), adhesive and migratory properties (19), as well as inflammatory potential (20). Trp metabolism has also been involved in various diseases ranging from chronic granulomatous disease (21) and gastrointestinal diseases (22) to neurodegenerative diseases (23). In obesity, IDO activity is up-regulated in adipose tissue, in circulating blood, and likely in the digestive tract of obese compared to non-obese subjects (24–28). The use of mouse models has recently involved its enzymatic activity in the pathogenesis of MetS (28). On the other side, administration of Kna, a metabolite downstream of Kyn, to WT mice has been shown to activate G protein-coupled receptor (GPR) 35, and increase energy expenditure (29), suggesting a protective effect. However, this may not represent the role of the endogenous Kna with physiological concentrations, as that experiment was performed in WT mice. It has to be noted that the mechanisms of IDO1 actions could be different in an autoimmune context such as type I diabetes, where the observed low level of IDO1 may weaken the immunomodulatory microenvironment, and make the pancreatic β-cells more susceptible to inflammatory deleterious response (30).

In the context of CVD, we previously showed that, in contrast with previous studies (31–34), IDO1 does not protect but rather promotes the development of atherosclerosis (35). We showed that IDO1 activity sustains an immune-stimulatory potential through inhibition of a major immune-regulatory and athero-protective cytokine, interleukin (IL)-10 (36). Consistently, it was shown that the induction of IDO1 by dietary microbial oxazoles reduced IL-10 production in intestinal epithelial cells (IECs). This mechanism was dependent on KP such as Kna, which inhibited IL-10 production through the activation of the aryl hydrocarbon receptor (AHR) (20). However, on the other hand, KP was previously shown to induce the generation of protective Tregs through AHR activation (37). AHR receptor is activated not only by small molecules coming from the host such as KP but also by environmental sources such as the diet, the gut microbiota, and pollutants (38). Evidence showed that AHR activation could have either pro- or anti-inflammatory effects depending on ligand- and/or cell-specific effects to locally modulate the inflammatory response (38). Moreover, it is important to know whether physiologically relevant concentrations of those ligands can activate AHR *in vivo*.

In human atherosclerotic lesions, we found increased levels of the IDO1-generated metabolite, Kna, which were associated with an unstable plaque phenotype. Moreover, Kna blood levels predicted death, and recurrent MI in patients admitted for acute MI (35). These results are in agreement with previous clinical studies showing that circulating Kyn and Kyn-derived metabolites are associated with cardiovascular risk factors (39, 40) and with worse outcome in patients with coronary artery disease (41–43). Collectively, these data indicate a potential involvement of IDO1 in MetS and CVD, and an emerging concept suggests that Trp catabolism through host expressing IDO1 in gastrointestinal tract could contribute significantly to cardiometabolic diseases.

## Tryptophan Metabolism in Gastrointestinal Tract

In the gastrointestinal tract, Trp metabolism has major effects on the host and notably on immunity and metabolism, gut microbiota, intestinal barrier, and transit (11). In mice, dietary lack of tryptophan leads to impaired intestinal immunity and promotes microbiota dysbiosis (44). Moreover, patients with intestinal bowel disease exhibit increased IDO activity, supporting the importance of Trp metabolism in maintaining intestinal homeostasis (22). In turn, gut microbiota derivatives could affect KP. Short-chain fatty acids (SCFAs), such as acetate and butyrate, are the end products of fermentation of dietary fibers by the anaerobic intestinal microbiota, and have been shown to exert multiple beneficial effects (7). Recently, it has been shown that butyrate negatively regulates IDO expression by IECs (45), suggesting a potential role of gut microbiota-derived metabolites in the regulation of Trp metabolism. Consistently, the absence of gut microbiota in germ-free mice was shown to reduce host IDO activity as assessed by decreased plasma Kyn/Trp ratio (46).

In homeostatic conditions, Trp metabolism in the intestine follows three major pathways: (i) the KP in both immune cells (mainly macrophages) and IECs via IDO1, which is the major pathway (represents 95% of ingested Trp). (ii) The direct transformation of Trp (4–6%) by the gut microbiota into tryptamine and indole metabolites via the action of the enzyme tryptophanase, which is expressed in many Gram–negative and Gram–positive bacteria. Among indole metabolites, indole pyruvic acid can give rise to indole propionic acid (IPA) and to indole acetaldehyde that can be converted to indole acetic acid (IAA), and then to indole aldehyde (IAld) (Figure 1). Some of indole metabolites such as IAA, indole-3-acetylaldehyde, IAld, and tryptamine have been shown to maintain intestinal barrier integrity and immune cell homeostasis through activation of the AHR (10, 22). Other studies demonstrate that intestinal barrier function can be improved by IPA through the activation of the pregnane X receptor (PXR). (iii) A small portion of Trp (1–2%) can give rise to the serotonin production pathway in enterochromaffin cells via Trp hydroxylase 1 (TpH1) (Figure 1).

**Figure 2:**
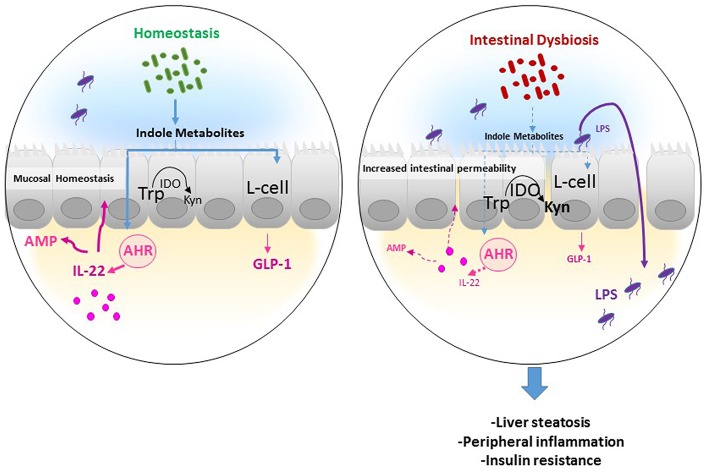
Potential mechanism of actions of Trp metabolites in the gastrointestinal tract. In homeostatic condition, Tryptophan (Trp) is used by the host indoleamine 2,3-dioxygenase (IDO) to be converted to Kynurenine (Kyn), and by gut microbiota to produce indole metabolites. Several indole derivatives have been described as activators of the AHR that promotes IL-22 production, which stimulates mucosal defense via the induction of antimicrobial proteins (AMP). In addition, indoles may affect mucosal homeostasis by stimulating mucin production and promoting epithelial barrier function by enhancing tight junction proteins. Also, indoles induce the release of the incretin, glucagon-like peptide 1 (GLP-1), in enteroendocrine L cells, which is known to stimulate insulin secretion, suppress appetite, and inhibit gastrointestinal motility, and secretion. In an inflammatory disease condition such as obesity, the increase in IDO activity leads to decreased indole production from Trp and thus low GLP-1 and IL-22 production leading to increased intestinal permeability and lipopolysaccharide (LPS) translocation in the systemic circulation, resulting in peripheral inflammation, liver steatosis, and insulin resistance.

The conversion of Trp to Kyn through IDO1 represents thus the major pathway of Trp degradation in the intestine, indicating the importance of the enzyme beyond Kyn in the pathway of Trp metabolism (Figure 2). Notably, we recently showed that high-fat diet supplementation was associated with increased IDO1 activity and inversely to a decrease in indole derivatives such as IAA, whereas IDO1 deletion leads to higher intestinal IAA production (28). In accord with mouse data, we observed a shift of Trp metabolism toward more Kyn and less IAA in feces of obese, and non-treated type II diabetic compared to non-obese subjects (28), suggesting an increase in gut IDO activity and a decrease in indole pathway in the context of MetS (Figure 2). The same was observed in the context of intestinal inflammatory diseases (22), suggesting the involvement of common mechanisms in diseases with disruption of intestinal barrier. Consistently, gut microbiota of individuals with MetS and with intestinal inflammatory disease showed decreased AHR activation (22, 47), suggesting that dysregulation of indole metabolites and AHR activation could be involved in inflammatory diseases.

Several bacterial Trp metabolites, such as indole metabolites, have been proven to be AHR ligands and to exhibit protective effects (48). Specifically, indole metabolites including indole, IPA, IAA, and tryptamine were shown to promote IL-22 production through AhR activation (10). IL-22 cytokine has been found to control epithelial cell proliferation and antimicrobial peptide (AMP) production, limiting the ability of commensal bacteria to cause inflammation, which has been shown in the context of MetS (Figure 2) (49), and more recently in atherosclerosis (50). Moreover, other studies demonstrate that intestinal barrier function can be regulated by indole derivatives, particularly IPA through activating the PXR, which inhibits inflammation locally and up-regulates tight junction expression (51). On the other hand, mouse models of obesity showed that peripheral serotonin produced by the gut favors MetS through a negative regulation of brown adipose tissue thermogenesis (52). However, this may be questionable in humans since the change of energy expenditure depending on brown adipose tissue in adult human is still under debate.

Indole absorption through the colonic mucosa is followed by its liver metabolism to indoxyl sulfate, the prototype of protein–bound uremic toxins. This “gut–liver axis,” driven by the local gut microbiota, could then exert peripheral effects. For example, in patients with chronic kidney disease, accumulation of indoxyl sulfate due to insufficient renal removal has been involved in CVD in these patients (53). However, this represents a supraphysiological concentration of indoxyl sulfate, which may not be representative to what happens in CVD patients without renal diseases. On the other hand, certain indole metabolites seem to exert anti-inflammatory effects (54). Future studies are needed to determine the role of indole metabolites, particularly indoxyl sulfate in cardiometabolic diseases without renal failure.

Gut Trp catabolism may have peripheral effects and could impact the development of cardiometabolic diseases. We recently showed that IDO1 deletion or inhibition in the context of MetS improved insulin sensitivity, decreased endotoxemia, and chronic inflammation, and positively regulated lipid metabolism in liver, and adipose tissues. We found that these beneficial effects were due to rewiring of Trp metabolism toward a microbiota-dependent production of IL-22 and were abrogated after treatment with a neutralizing anti-IL-22 antibody. Moreover, microbiota transfer of feces from obese mice treated with IDO1 inhibitor (L-1 methyltryptophan, L-1MT) compared to non-treated mice increased IAA as well as IL-22 production and improved metabolic parameters in the recipient mice fed with high-fat diet (28). In addition, we and others have recently shown that indole metabolites and particularly IAA protect against MetS complications (28, 47), highlighting the importance of IDO1 activity on intestinal homeostasis and peripheral metabolism. The observed protective role of indoles may be related to their local effects on intestine through promoting IL-22 production and/or the stimulation of enteroendocrine L cells to produce glucagon-like peptide-1 (GLP-1), an incretin stimulating the secretion of insulin by pancreatic β cells (47, 55). Moreover, indole was shown to alleviate liver inflammation in mice through preventing LPS-induced detrimental effects (56). In this context, the supplementation with Lactobacillus strain bacteria stain producing high levels of indole metabolites leads to improvement of metabolic parameters, through maintaining intestinal barrier function and promoting GLP-1 production (49).

## Conclusion and Perspectives

Understanding the relationships between the diet and the complex cross-talk between the host and gut microbiota appears now as instrumental for the development of new therapeutic approaches to modulate metabolic dysbiosis and treating disease. Data demonstrate the importance of indole metabolites in the re-establishment of intestinal epithelial barrier integrity in the context of intestinal inflammatory diseases and MetS. However, because different bacteria may possess diverse catalytic enzymes, it is complicated to predict which indole metabolites are produced and which ones may activate AHR. Thus, the physiologic implications of AHR activation by tryptamine and the different indole metabolites in the gastrointestinal tract remain to be established. Also, future studies are needed to determine their involvements in co-associated diseases such as CVD. Although several bacteria capable of producing Trp catabolites such as lactobacilli have been identified, others may exist and should be identified through, for example, the use of bacterial metabolome approaches. One major achievement would be to identify the best way to modulate Trp catabolism to improve dysbiosis and metabolic health. For example, further studies are warranted to investigate whether enhancing indole derivative formation by bacteria or directly administering those metabolites is beneficial in inflammatory diseases characterized by gut barrier disruption. Also, future studies are required to determine therapeutic efficacy of whether these bacteria and/or indole metabolites should be used in a combined or separate manner.

The use of IDO inhibitor failed to show efficiency in cancer; one possibility is to reuse this inhibitor in patients with metabolic diseases. In this context, the development of other IDO inhibitors, for example, oral non-absorbable drugs, to inhibit locally IDO in gastrointestinal tract thereby enhancing effectiveness while reducing potential side effects, would be valuable. However, before the development of such potential treatments, a more comprehensive and mechanistic understanding of factors that influence the different Trp catabolism pathways is critical to elucidating the physiologic functions of IDO and the consequences of its disturbance.

## Author Contributions

The author confirms being the sole contributor of this work and has approved it for publication.

### Conflict of Interest Statement

The author declares that the research was conducted in the absence of any commercial or financial relationships that could be construed as a potential conflict of interest. The handling editor declared a shared affiliation, though no other collaboration, with the author ST.
